# New Edition of the London Pharmacopœia

**Published:** 1808-08

**Authors:** 


### *New Edition of the London Pharmacopœia.*

THE Committee appointed for the revision of the Pharmacopœia have submitted a specimen to the Fellows, Candidates, and Licentiates of the College, and solicited upon an object of such general concern and importance, the opinions and suggestions of each individual to whom the specimen is addressed.

In the progression, they observe, of human knowledge, pharmacy cannot remain stationary, and the College have accordingly accommodated it to existing circumstances, at suitable intervals, and thereby regulated and improved the practice of medicine in this country. Such a revision they have felt themselves called upon to make, at the present time, by the vast improvement in the several branches of science, with which pharmacy is more especially connected, since the year 1787, and they think it proper to state generally the principles upon which various alterations have been adopted in the present instance.

These alterations are referable to the several heads of nomenclature, weights, and measures, arrangements, processes, the omission of former articles, and the introduction of new ones.

To each of these it will apply as a general observation, that practical application and convenience have been assumed as fundamental points, which the Committee have endeavoured constantly to keep in view.

1. *Nomenclature.* At the time of the publication of the last Pharmacopœia, modern chemistry was in its infancy; its language, (which professed to describe, and not merely to designate a substance by its name), was new in principle, and the application of it not generally received. Various terms, therefore, of that Pharmacopœia differ essentially from those which have since been established in the Science, and it has been incumbent upon the Committee to consider in the present instance, whether the nomenclature of chemistry might be still farther and more minutely adopted. As far as arbirtary names (to which common consent has affixed precise ideas) go, and also in compounds consisting of two ingredients only, or where different proportions of the same constituent parts are to be expressed, it has been thought proper to receive those terms which general chemistry employs; but as a large proportion of pharmaceutical preparations consist, strictly speaking, of more complex combinations, which cannot be expressed correctly without periphrasis and inconvenience, and are therefore but ill suited to the purposes of prescription, the Committee have judged it sufficient to designate these, without attempting at the same time to describe their composition; and whether the name has been drawn from some circumstance of preparation, or quality, they have cautiously endeavoured to make such distinctions as may be least liable to error in the ordinary method of practice, and may not contradict the received chemical doctrines, or mislead in their application.

The names of vegetables have also been accommodated to the latest systems of Botany, so that they may not hereafter contradict the terms of that science, or deceive the practitioner in his references thereto. Many names of medicinal plants were in the earlier periods of Botany drawn from those of families to which modern system does not admit them to belong,* but have been retained in pharmacy, though wholly at variance with the improved state of science. The committee trust they have been able to remedy this inconvenience without very frequent violence to the names commonly employed. They have thought it most convenient, and fully sufficient, to experess each article in general by a single word,† and have retained the former one wherever it accorded either with the generic or specific name of Linnæus, both of which, however, it has been necessary to employ, for the purpose of distinguishing between them, when more than one species is taken from the same genus.‡ There being some vegetable substances, the names of which are in a manner independent of botanical nomenclature,§ no alteration with respect to these seemed necessary, for in fact they are not at variance with modern science. Intending, moreover, that the pharmaceutic name shall, where a part of a plant is

* Cicuta. Helleborus albus.

† Aconitum. Cascarilla

‡ Rosa Gallica. Rosa canina.

§ Arabicum Gummi.

is used, refer to that part only, they have transferred the term expressive of such part from the first column of the catalogue, in which it formerly stood, to the second.

2. *Weights and Measures.* From the great uncertainty of the customary mode of dividing by drops any quantities of liquids of less bulk than a drachm, and the increase of that uncertainty by the late introduction into some shops of measures applying to liquids of different densities, the bulk of a drop of water as a standard, the Committee have been led to consider the subject more particularly, and to adopt means for the removal of this uncertainty in the exhibition of many active remedies for the future. They have, for this purpose, adopted the gradulated measure of the late Mr. Lane, which is founded upon an accurate division of the exchequer wine gallon down to the $\frac{1}{60}$ part of a drachm, and which is equivalent to a drop of water. Of course it is their intention, that the common method of dropping liquids of different densities should be disused, and the measure received into the shops of apothecaries, a point upon which it will be necessary to place especial stress, in order that prescriptions may be accurately prepared. As the same Latin term has been employed to express the pint measure and the pound weight, they have extended the same resemblance to inferior measures, and have the more readily substituted *granum* for *gutta*, because the latter term implies that peculiar mode of division which they wish to deprecate.

3. *Arrangement.* On this head, it is only necessary to observe, that the chapters have been arranged in what appeared to be a more natural and convenient order of the substances concerned than the former one.

4. *Processes.* Considerable alteration has been made in various processes, by which the Committee hope they will be found more accommodated to general use. Expence in preparation ought not to be balanced against correctness and uniformity, and it is to be lamented that the profits and competition of trade should have induced a very extensive disposition to deviate from the directions of the Pharmacopœia. To this point, therefore, the Committee have looked with much attention, and, as far as they have thought themselves justified, they have endeavoured to make such deviation less an object to the operating Chemist than heretofore; for this purpose they have not looked in their formulæ to that accuracy which would be necessary for chemical tests, but rather to the uniformity of the preparation, and its use as a medicine. The directions for manipulation manipulation are given *generally*, because they admit of some variety in their application in many instances, according to the scale on which they are prepared, and other circumstances; the Committee trust, however, that, if their directions be followed, the results will be, in the same proportion, uniform and correct, and that the well educated Apothecary will have no difficulty in understanding and applying them. Under this head, it is particularly incumbent upon the Committee to acknowledge the great advantage they have derivrd from the liberal communications of the Society of Apothecaries, with respect to the practice of their extensive concern, and also from many individuals engaged in chemical preparations upon a large scale.

5. *Omission of former Articles and Introduction of new ones.* In the rejection of many substances of trifling importance or efficacy, of others which have appeared rather to belong to extemporaneous prescription, and of certain forms of medicine which have become obsolete in general practice, and also in the introduction of any new articles, the Committee have exercised their own judgment freely, but they trust with sufficient caution. They hope the College at large will approve of their having neglected to insert many substances which individual practitioners have recommended and employed, where such have not received the sanction of more general experience. They conceive further, that a strict examination of its powers ought to precede the introduction of any article into the Pharmacopœia, and that the late appointment of a Committee of the College for this express purpose will hereafter appreciate the value of such recommendations, by surer tests than those which have heretofore been deemed sufficient.

It is scarcely necessary they say to state, that the present Specimen merely submits to the College the result of the deliberations of a Committee, and that neither their attention or knowledge can be supposed to have comprised therein all the circumstances which deserve consideration; the Committee, therefore, await those corrections which the experience and observation of the profession at large may communicate, and which it will be their duty to consider, and estimate attentively, as they proceed; and they trust that, in its final arrangement, the new Pharmacopœia will contribute to the honor of the College, and an improved practical application of the Science of Medicine.

The Committee request that the copy of the Specimen sent to every Member of the College may be returned, with any remarks thereon, under cover, to the *Register of the*

*the Royal College of Physicians, Warwick-lane, London,* on or before the 25th of July next.

The following specimen of the new names will give our readers an idea of the Nomenclature intended to be adopted, Ammonia carbonica, liquor ammoniæ, potassa carbonica, soda carbonica, potassa sulphurica, soda phosphorica, potassa sulphurata, arsenicalis liquor, (Fowler's solution) ferrum muriatico-ammoniatum, ferrum sulphuricum, &c.

On some occasions the Committee have designated the same substance by different names, e. g. corrosive sublimate is called hydrargyrus corrodens muriaticus, and hydrargyrus oxi-muriaticus, rhubarb both rhabarbarum and rheum, calomel is called hydrargyrus mitis muriaticus. Many of the old names are retained, as pulvis antimonialis, vinum ferri, cerussa acetata, decoctum cinchonæ. Amongst the new infusions we find those of cloves, cascarilla, colombo, quassia, rhubarb, simarouba, tobacco, catechu, digitalis, and tar.

Among the new extracts occur those of the hop, taraxicum, aconite, belladonna, and rhubarb.

The musk mixture is retained, and the saffron is proposed to be still kept in the compound tincture of aloes, and the tinctures of rheum, capsicum, digitalis, and hops are proposed; and we would recommend a tincture of conium as the extract does not keep well. We conclude that the syrup of saffron is retained on account of its colour; and probably the same motive has induced the Committee to retain it in the aromatic confection and compound tincture of bark, though it has long been known to possess no medicinal virtues.

As opium is the most important article in the Materia Medica, and as many patients entertain an insuperable prejudice against it, we should have rejoiced to have seen both liquid and dry preparations of it, in which the *name* is suppressed, as it is in the pulv. ipec. comp. and in the tinctura camp. comp. We have no doubt that an elegant and useful tincture or solution of opium, in imitation of the well-known black drop, would be a valuable addition to the new Pharmacopœia.

*Directions*

